# Ethanol leaf extract of *Hoslundia opposita* in *in vivo* antioxidant and hepatoprotective activity using an animal model

**DOI:** 10.37796/2211-8039.1321

**Published:** 2022-09-01

**Authors:** Fred Coolborn Akharaiyi, Odoligie Imarhiagbe, Lucky Efe Isunu, Adebayo Thomas Ajibola

**Affiliations:** a Department of Microbiology, Edo State University Uzairue, KM 7 Auchi-Abuja Road, Iyamho-Uzairue, Edo State, Nigeria; b Department of Biological Sciences, Edo State University Uzairue, KM 7 Auchi-Abuja Road, Iyamho-Uzairue, Edo State, Nigeria; c Department of Microbiology, Federal University of Technology, Akure, Ondo State, Nigeria; d College of Health Science and Technology, Ijero-Ekiti, Ekiti State, Nigeria

**Keywords:** Animal model, Extract, Hepatic protection, *Hoslundia opposita*, Toxicity

## Abstract

**Introduction:**

The liver is a valuable organ responsible for detoxifying harmful substances from the body. It plays an essential role; hence the need to ensure its protection from damages. The management of liver diseases with orthodox medicine has been found to have side effects; consequently, there have been several calls on the use of alternative medicine for the effective management of liver diseases.

**Aim:**

This study aimed to evaluate the toxicity and antioxidant potentials of *H*. *opposita*.

**Materials and methods:**

The leaves of *H*. *oppisota* harvested from a forest were processed and extracted with ethanol. The extract concentrations of 100–400 mg/ml were used to evaluate acute toxicity, biochemical analysis, *in vivo* antioxidants, and histopathology using an animal model.

**Results:**

The acute toxicity test of *H*. *opposita* ethanol leaf extract studied for 21 days suggested safety at a concentration of 400 mg/kgbw. Weight gain in the negative control was 16.91 g, while weight loss in the positive control mice was (13.96 g). 400 mg/kg was found as the LD50 of the plant extract. A decrease in uric acid, cholesterol, urea, creatinine, and bilirubin contents was observed in the single extract-treated mice and the paracetamol-induced but co-administered with extracts, while increased values were observed for protein and albumin contents. The positive control values of ALT, AST, and ALP were 66.74 ± 3.51 IU/L, 68.52 ± 3.63 IU/L, and 342 ± 3.04 IU/L, respectively, in the negative control, values were 48.16 ± 3.68 IU/L, 37.46 ± 1.52, and 89.34 ± 2.66 IU/L. There was a reduction in lipid peroxidation in the extract-treated and satellite groups. At the same time, increased values were observed for catalase and glutathione biochemical activities. The effects of a high dose of paracetamol were alleviated by the ethanol leaf extract over time.

**Conclusion:**

The irregularities in the *in vivo* biochemical, *in vivo* antioxidant values, and the hepatic damages caused by paracetamol toxicity were regulated on extract treatments, suggesting its use traditionally for the treatment of liver diseases.

## 1. Introduction

The liver can be affected by some conditions and diseases, making it malfunction in its reliability in human health. The liver, a vital organ in the body, serves many critical functions, of which its deficiency can lead to severe damages to the body or even death. Once the liver is injured or diseased, nutrients and chemicals processing and filtering before general circulation into the body system will be limited. Protein production, glycogen, cholesterol, and some other products the liver requires for normal body activities can also be hampered to promote ill-health in humans. The high rate of liver disease is primarily problematic in developing countries, and liver disease has resulted in morbidity and mortality worldwide. Liver infections are frequently caused by hepatitis A, B, and C, viruses, carbon tetrachloride (CCl4), and even high doses of paracetamol [1,2]. As threatening as liver diseases, they can be managed with medicinal plants rich in antioxidants to prevent or delay manifestation and cure. Several researchers have proven this with active chemical compounds in some plants [3,4]. Such phytochemicals are flavonoids, tannins, steroids, anthocyanin, saponins, and even vitamins. Antioxidants in the biological system may be enzymatic or non-enzymatic. Enzymatic antioxidants are superoxide dismutase, catalase, and glutathione, while the non-enzymatic antioxidants are polyphenols, vitamin C, vitamin E, selenium, and carotenoids. These types of enzymatic antioxidants are responsible for neutralizing several free radicals [5], resulting in liver damage.

*Hoslundia opposita* is a perennial and shrub-like flowering plant found in the wild or grown for its medicinal importance in many sub-Saharan countries of Africa. The plant has antipyretic, diuretic, and antimalarial properties. *H*. *opposita* Vahl is classified under the kingdom of Plantae, family: of *Lamiaceae* [6,7]. Phytochemicals screened from this plant are tannins, steroids, flavonoids, terpenoids, saponins, and glycosides. Some compounds, including euscaphic acid, hoslunddol, ursolic, and 5,7-dimethoxy-6-methylflavone, have been isolated from the leaves of *H*. *opposita*. These critical chemical compounds could be responsible for the plant’s leaves’ physiological activity. The use of *H*. *opposita* for ethnopharmacological and ethnobotanical has suggested the aim in this study to evaluate its toxicity and antioxidants potentials.

## 2. Materials and methods

### 2.1. Collection and preparation of plant sample

Fresh and healthy-looking leaf samples of *H*. *opposita* was collected from the forest in Iyamho community, Uzairue, Edo State. The leaf samples were harvested in July 2020 and was authenticated by Dr. Imarhaigbe Odoligie of the Biological Science Department, Edo State University Uzairue. The plant sample with identification number A426EUI was deposited in the Departmental herbarium.

The collected leaves were rinsed severally in clean water and shred-dried at room temperature for three weeks. The dried leaves were pulverized in a mechanical grinder to obtain a smooth powder. 400 g of the powder was weighed and extracted with ethanol at room temperature for 24 h. The extract was filtered and concentrated in-vacuo to semi-solid. The extract was kept in a sterile bottle for use.

### 2.2. Acute toxicity test

*In vivo* toxicity of the extract was performed using the criteria of WHO [8], OECD, [9]. Fifteen Swiss albino mice were used to estimate LD50 by dosing the animals orally. Before the test, the animals were initially fasted for 5 h and divided into groups of five with three animals each for dose level of normal saline (control group) and 100, 200, 300, and 400 mg/ kgbw (treated groups), respectively. After the treatment, as described by Lorke [10], toxic symptoms were observed on the mice for twenty-one days to cater to any delayed effect of the extract concentrations. Estimation of LD50 of the extract concentrations was by the geometric mean of death caused by lowest dose and no death by highest amount using the below formula:


LD50=√(A×B)

Where A = the maximum dose that produces 0% death and B = the dose that has 100% death according to Lorke [10], the LD cut-off for the extract concentrations was 450 mg/kgbw, thus the therapeutic extract dose for the study was between 100 and 400 mg/kg.

### 2.3. Experimental animals

Swiss albino mice (*Mus musculus*) with body weights of between 23 and 34 g were quarantined for two weeks. The mice were allowed to feed on standard rat pellets and water. After that, the mice were conducted according to the NIH guide and denied feed for 16 h but given water only. Health Research Ethical Committee approved the experimental procedures performed on the animals with the given number of NHREC/08/2016.

### 2.4. Experimental design

Six mice each were conducted in a group. Group, I mice were fed with rat pellets and water for negative control. Group II mice were dosed with 1 g/kgbw of paracetamol three times daily at 8-h intervals for three days (positive control), while groups III to VI were treated for seven days with extract concentrations of 100, 200, 300, and 400 mg/kg BW respectively after a thrice-daily dose of 1 g\ /kg of paracetamol for three days.

### 2.5. Biochemical analysis

Total albumin was experimented by the method of Doumas et al. [11], uric acid was determined by the criteria of Carroll et al. [12], total protein by the process of Hutson et al. [13], urea determination as described by Fenech and Tommasini [14], estimation of bilirubin as described by Watson and Rogers [15], for cholesterol, by the described method of Abel et al. [16] and creatinine level was performed using the technique of Lustgarten and Wenk [17].

### 2.6. In vivo antioxidant activity of the extract

This method was used to determine the total antioxidant capacity of *H*. *opposita* leaves extract translated into the mice tissue. The obtained liver organ from the experimented mice was washed severally with 10% saline (w/v) and homogenized in cold 0.01 M phosphate buffer at a pH of 7.4. Supernatant from the homogenate was obtained by centrifugation for 1 h at 10000 g. The supernatant was collected and used to assay for antioxidants by the criteria of Van Der Sluis et al. [18]. Lipid peroxidation level and reduced glutathione level were measured by adopting the method of Ellman [19] and catalase by Cohen et al. [20].

### 2.7. Histopathology of liver tissues

The Liver samples of the experimented mice were collected and washed with normal saline. The samples were cut into small sizes of about 2 cm and dehydrated in grades of ethanol. After dehydration, xylene was used to clear traces of ethanol and water from the tissues before impregnating them in paraffin wax for 1 h at a controlled temperature of 60 °C. The tissues were then embedded in molten paraffin wax and sectioned with a microtome (Bright, England) at 4–6 μm. The sectioned tissues were floated in a water bath regulated at 35 °C and picked with slides that were previously robbed with egg albumin and allowed to dry at room temperature. The tissues were then de-waxed with xylene, hydrated, clear with xylene, stained with hematoxylin and eosin, and mounted with DPX. The prepared slides were allowed to air-dry and photographed. The pictures were then observed with a binocular microscope for a level of damages or safety.

### 2.8. Statistical analysis

On a statistical basis, results were presented as mean ± Standard error of means. Differences in one group to other groups were compared by One-way Analysis of Variance (ANOVA) followed by the Duncan Multiple Comparison Test using SPSS version 16.

## 3. Results

The acute toxicity test of *H*. *opposita* ethanol leaf extract suggests safety for use in this study at concentration of 400 mg/kgbw. From a dose of 50–400 mg/kgbw, there was no record of death in the population of mice during the 21 days toxicity test. Even as no mortality was recorded, none of the mice showed any sign of clinical pathology for the 21 days of the experimental study. By this, 400 mg/kg was confirmed as the LD50 of the extract (Table 1). While there was an increase in weight for 21 days study among the extract-treated and negative control groups, a decrease in weight was encountered in the positive control group. Weight gain with 100 mg/kg for the period of study was 8.97 kg/dL, with 200 mg/ kg it was 6.05 kg/dL, with 300 mg/kg, it was 3.89 kg/ dL and with 400 mg/kg, weight gain was 3.07 kg/dL. Weight loss for 21 days study in positive control mice was (13.96 g), while in the negative control, weight gain was 16.91 g (Table 2).

The *in vivo* biochemical test of the induced mice with paracetamol and co-administered with single extracts suggests a decrease in mice’s uric acid in extract concentration. During this study, uric acid decreased from 9.02 ± 0.13 mg/dL to 8.35 ± 0.76 mg/ dL in the single extract-treated mice of 100–400 mg/ kgbw, while it was from 9.43 ± 0.25 mg/dL to 8.55 ± 0.32 mg/dL in the paracetamol-induced but co-administered with extracts. Cholesterol, urea, creatinine, and bilirubin contents also decreased in values on extracts treatment. Significant (p < 0.05) differences existed between the satellite group and the negative control in all these parameters. While these observations of decreased values, total protein and albumin values increased on extract concentration bases. Total protein in the satellite groups with 100 mg/kgbw increased from 7.23 ± 0.22 to 7.63 ± 0.23 mg/dL with 400 mg/kgbw. Total albumin increased by 1.08 mg/dL between 100 mg/kgbw and 400 mg/kgbw (Table 3).

Table 4 illustrates the *in vivo* biochemical activities of the tested extracts. Higher values in ALT, AST, and ALP were observed in the positive control than in the negative control group of mice. However, decreased values on extract concentration bases were observed in the single extract dose and satellite groups of mice. The decrease in values perhaps becomes normal; hence, it indicates healing affinity, thus values tilting towards the negative value as recovery.

The *in vivo* antioxidant quality of the extracts is presented in Table 5. There were significant (p < 0.05) differences between the tested parameters’ positive control and negative control values. In lipid peroxidation, a decrease of values in both the extracts treated and satellite groups, while increased values were recorded in the catalase and glutathione biochemical activities. The decreased and increased values recorded in these *in vivo* antioxidant quality parameters balanced with the negative control values of the various parameters investigated. There was no effect of the extract to deduce a significant (p > 0.05) difference in the bodyweight of treated mice compared with the negative control group.

**Fig. 1:**
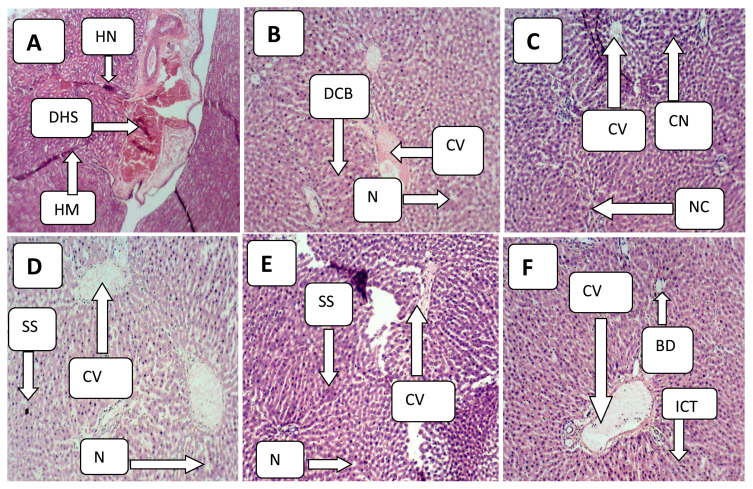
Photomicrograph of sectioned liver tissues: A = Representative of positive control group showing hemorrhage (HM), distorted hepatic structure (DHS) and Hepatic necrosis (HN). B = Representative of negative control group showing normal architectural structures of distinct cellular boundaries (DCB), Central vein (CV). C = Representative of paracetamol-induced but co-administered with 100 mg/kg extract showing necrosis (NC), Clump nucleus (CN) and Central vein. D = Representative of paracetamol-induced but co-administered with 200 mg/kg extract showing cellular infiltration (CI), sinusoid (SS), E = Representative of paracetamol-induced but co-administered with 300 mg/kg extract showing central vein (CV), Sinusoid, nucleus. F = Representative of paracetamol-induced but co-administered with 400 mg/kg extract showing central vein (CV), interlobular connective tissue (ICT) and bile duct (BD).

The experimented liver tissues of the positive control group mice showed some distorted morphological structures such as focal necrosis, dark spots, irregular surfaces, necrotic lesions, and infiltration, among others that confirmed paracetamol effect on the mice livers which were differentiated from the negative control group of mice with typical architectural structures. However, the histopathological examination also confirmed that recovery from hepatic damages caused by paracetamol was possible with the co-administered ethanol extracts of *H. opposita* (Fig. 1).

## 4. Discussion

*H*. *opposita* plant plays several roles in traditional medicine due to its active chemical compounds. For instance, Köhler et al. [21], Mohammed et al. [22], Salia et al. [23], Sadri [7] have reported on its efficacy for treating various ailments. Based on these, the necessity of its acute toxicity in the *in vivo* biochemical and *in vivo* antioxidant activities was investigated and determined the hepatoprotective potential of ethanol leaf extract of the plant.

On dosing the mice with the prepared extracts, the injected leaf extract concentrations into the system of the mice served as bases to interpret the safety and the health potential of the leaf extract concentrations. Mortality of mice with the extract concentration of 400 mg/kgbw was not feasible, thus LD50 of the extract and the choice of using concentrations of 100–400 mg of extract in this study.

The mice weight gain of the negative control compared with the positive control suggested abnormality in the mice system as caused by the damages elicited with the induced-paracetamol. Evidence has shown from several kinds of literature that paracetamol overdose can result in liver dysfunction [24]. The weight gain in the extract-treated mice after inducement with paracetamol suggests the extract might improve digestive health and other physiological activities beneficial for sound health and growth. Green vegetables are vital minerals, fibre, essential fatty acids, amino acids, and vitamins [25]; others include the bioactive ingredients responsible for health promotion, such as phenolic and antioxidant compounds [26–28]. These could be responsible for the quick absorption in the mice to gain much weight even as it is a proven fact that green vegetables play an active role in indigestion. This is accomplished by helping hormones break down foods to move through the system quickly. Administration of the leaf extract at different concentrations showed no level of significance (p < 0.05) increase in weight of rats, and this observation can be correlated to the study of Emeka and Osundiya [29]. The statement indicates that the employed concentrations of the plant extract were helpful to the mice in terms of vital nutrient supply. It could also be regarded as a vegetable with potential hepatoprotective properties based on its composing biochemical activities and *in vivo* antioxidant potentials.

Biomarkers served for the determination of hepatic damages. Evidence denoting this in the study is the shift of protein level from the standard unit, which was reflected in functional changes in the livers that consequently affected the overall physiological function in the mice. Also to be considered is the reduction in glutathione which indicated suppression of Reactive Oxygen Species (ROS) by free radical scavenging and antioxidant potentials of the ethanol leaf extract of *H*. *opposita*.

Ordinarily, in a standard dose of paracetamol, the active metabolite – N-acetyl-p-benzoquinone imine (NAPQI) is usually detoxified by conjugation with reduced glutathione and is eliminated through the kidney [30]. But with the excessive dosage of paracetamol-induced into the mice, glutathione could not detoxify NAPQI, resulting in liver damage. Damages caused to the liver with a high dose of paracetamol have been reported by several authors [31]. The use of paracetamol in overdose becomes necessary to screen for *H*. *opposita*’*s* ability as a hepatoprotective herbal medicine. Several authors have researched herbal medicine as hepatoprotective agents against paracetamol-induced laboratory animals [29,32,33].

Biomarkers of uric acid, cholesterol, urea, and bilirubin were higher in the positive control than the negative control. At the same time, creatinine, total protein, and total albumin values were more elevated in the negative control than in the positive control. The higher values of uric acid, cholesterol, urea, and bilirubin in the positive control than the negative control were basically on hepatic, myocardial, and renal dysfunction in the mice. In contrast, the higher creatinine, total protein, and total albumin values in the negative control than in the positive control are connected to the liver enzyme function and integrity. These enzyme markers were possible in circulation because of the damages caused by paracetamol to the liver tissues, resulting in various alterations in biochemical indices. Also, the overdose of paracetamol administered increased the biochemical enzyme markers of ALT, ASP, and ALP. Outside it, these are marker enzymes for liver function; their high manifestation is mainly during acute toxicity but is decreased in measures in prolonged intoxication due to liver damage [34]. The prominent appearance of these biomarkers in circulation determines the level of liver cell damage. However, treatment results with *H*. *opposita* extract showed recovery from injury as AST and ALT measures in serum were of lesser leakage in circulation, which could be related to free radical scavenging potentials the plant extract possesses. The reduced values in ALT and AST of the liver serum after treatment with the ethanol leaf extract prove recovery of the liver from injury, thus partially performing its ordinary activities for the mice’s health status. It could also be considered that the increased ALP level and decrease in bilirubin of the liver serum after extract treatment suggests recovery from injuries to confirm the ethanol leaf extract of *H*. *opposita* having a hepatoprotective agent.

The decrease in protein value observed is linked to its poor synthesis due to injury sustained by the liver with NAPQI from an overdose of paracetamol. This depletion in protein suggests that the poly-ribosome has been dissociated from the endoplasmic reticulum. Other reasons for the decrease in total protein level could be a result of the liver being the most excellent chemical factory in the body not able to build complex molecules from simple substances absorbed from the digestive tract and unable to manufacture bile which aids fat digestion and synthesis of protein. Total protein content level that was drastically decreased in the positive control mice suggests degradation of protein which led to the increase in hepatic damage that resulted in the severe liver necrosis observed in the liver histopathology. Bilirubin is toxic, and it is removed from the body system by albumin, which carries it to the liver to detoxify. The breaking down of hemoglobin by the overdose of paracetamol produces sufficient injury to hepatic parenchyma to cause a significant increase in the bilirubin content. Because of the damage sustained by the liver, it was unable to process albumin, which invariably increased the level of bilirubin in circulation. The high increase level of these enzyme markers in the circulation of the positive control mice, which were of low values in the negative control mice, further confirmed that the damages observed in the positive control mice resulted from the damages incurred with an overdose of paracetamol. Urea level was also increased in the paracetamol-induced mice. This indicates that pathological changes resulted in the liver. At the same time, this was pertinent; reduction in urea level of the satellite group where pretreatment with the plant extracts also suggested that the liver average functional ability was altered. The plant extracts, able to reduce the high urea level due to paracetamol injury, means it is a hepatoprotective agent.

Measures of lipid peroxidation (LPO), catalase (CAT), and glutathione (GSH) had shifted from standard units in induced mice with an overdose of paracetamol. This was abnormal, mainly where a higher value in LPO was recorded in the positive control group than the negative control group of mice; and lower values in CAT and GSH in the positive control group than the negative control group of mice. The body system of mice experienced toxicity from the induced excess paracetamol as a disputant free radical that results in oxidative stress. The antioxidant system of mice was unable to prevent the sudden occurrence of the excessive free radicals, thereby reducing the glutathione and elevation of the LPO found in circulation. Yousef et al. [35] reported that LPO and oxidative stress are relevant factors worth considering in hepatotoxicity caused by paracetamol. The lower measures of CAT and GSH in the positive control and the extract dosed mice than the negative control group of mice could result in the increase of LPO in the liver and dysfunction of mitochondrial and liver necrosis [36]. Hinson et al. [30] reported that GSH in a reduced measure is responsible for endogenous antioxidants, and it can counterbalance damages caused by free radicals. Overdose of paracetamol led to the decrease of glutathione in the liver serum, resulting in the liver’s malfunction for bilirubin processing and albumin production. This can result in the many physiological abnormalities observed in the mice.

Invariably, where abnormalities in the *in vivo* antioxidant and *in vivo* biochemical status in mice after paracetamol inducement and extract initial dose, the satellite groups of mice make understood the efficacy of *H*. *opposite*. Interpretation of the results emphasized on experimented parameters showed adjustment in the liver injuries for the certainty of physiological indices towards the health status and regular functions in the mice system.

It could be considered too that the increased ALP level and decrease in bilirubin of liver serum after extract treatment suggests recovery from injuries to ascertain the ethanol leaf extract of *H*. *opposita* having a hepatoprotective agent.

## 5. Conclusion

In this study, the ethanol leaf extract of *H*. *opposita* was reasonably safe at 400 mg/kgbw of the mice. The safety was deduced from the mice’s health status and weight gain at the acute toxicity test. The shifts in the *in vivo* biochemical values, *in vivo* antioxidant values from standard units, and the hepatic damages caused by paracetamol toxicity but regulated with extract treatment are considerable for the plant to have an active hepatoprotective agent. This has then provided evidence for its traditional use in treating liver diseases. Invariably, the created teamwork of the *in vivo* antioxidant potential and the plant’s extract hepatoprotective ability stands in the reduction of paracetamol-induced liver damage.

## Figures and Tables

**Table 1:** Acute toxicity evaluations of H. opposita in rats.

Extract dose (mg/kg)	Rate of death after 24 h exposure
50	0 out of 4
100	0 out of 4
200	0 out of 4
350	0 out of 4
400	0 out of 4
LD_50_	≥400 mg/kg^bw^

**Table 2:** Weight gain of mice during extract treatment.

	0 Day	7th Day	14th Day	21st Day
Negative control	25.16 ± 0.4	30.20 ± 0.0	34.13 ± 1.4	42.07 ± 0.8
Positive control	30.07 ± 0.7	24.12 ± 1.6	22.06 ± 0.5	16.11 ± 0.3
100 mg/kg	24.12 ± 0.1	28.17 ± 1.2	30.01 ± 1.3	33.09 ± 1.2
200 mg/kg	26.05 ± 0.5	29.12 ± 0.5	31.11 ± 0.7	32.10 ± 0.4
300 mg/kg	28.15 ± 1.3	30.06 ± 1.8	31.13 ± 0.1	32.04 ± 1.2
400 mg/kg	30.09 ± 1.3	31.21 ± 0.5	32.08 ± 1.5	33.16 ± 0.2

Values are mean ± standard deviations of triplicate determination.

**Table 3:** H. opposita ethanol leaf extracts on liver functions of mice.

Group of mice	Uric acid (mg/dL)	Cholesterol (mg/dL)	Urea (mg/dL)	Creatinine (mg/dL)	Bilirubin (mg/dL)	Total protein (mg/dL)	Total albumin (mg/dL)
Control (−)	7.01 ± 0.42	111.86 ± 0.54	19.24 ± 0.17	2.08 ± 0.25	1.11 ± 1.12	8.06 ± 0.31	4.96 ± 0.18
Control (+)	9.12 ± 0.36	222.06 ± 0.43	24.25 ± 0.27	1.89 ± 0.16	2.34 ± 0.21	6.24 ± 0.87	1.78 ± 0.42
100mg/kg^bw^	9.02 ± 0.13	170.23 ± 5.22	24.04 ± 0.36	1.80 ± 0.11	1.86 ± 0.32	7.18 ± 0.12	1.98 ± 0.11
200mg/kg^bw^	8.76 ± 0.24	162.03 ± 7.03	23.18 ± 0.27	1.62 ± 0.52	1.72 ± 0.16	7.11 ± 0.56	2.06 ± 0.43
300mg/kg^bw^	8.42 ± 0.36	157.13 ± 3.06	23.04 ± 0.21	1.47 ± 0.26	1.67 ± 0.11	6.52 ± 0.31	2.18 ± 0.18
400mg/kg^bw^	8.35 ± 0.76	153.24 ± 1.33	22.15 ± 0.35	1.40 ± 0.21	1.62 ± 0.35	6.87 ± 0.24	2.52 ± 0.40
PCM+100mg/kg^bw^	9.43 ± 0.25	315.04 ± 0.54	27.51 ± 0.22	3.04 ± 0.22	2.36 ± 0.14	7.23 ± 0.22	3.18 ± 0.01
PCM+200mg/kg^bw^	9.26 ± 0.11	301.24 ± 0.44	27.35 ± 0.52	2.18 ± 0.21	2.22 ± 0.42	7.27 ± 0.14	3.23 ± 0.72
PCM+300mg/kg^bw^	8.73 ± 0.24	215.31 ± 0.18	25.12 ± 0.41	1.66 ± 0.34	1.55 ± 0.31	7.45 ± 0.18	4.05 ± 0.18
PCM+400mg/kg^bw^	8.55 ± 0.32	184.18 ± 1.25	23.16 ± 0.11	1.38 ± 1.06	1.46 ± 0.38	7.63 ± 0.23	4.26 ± 0.25

Values are mean ± standard deviations of triplicate determination.

PCM = Paracetamol co-treated with extract concentrations.

**Table 4:** Effect of H. opposita on biomarkers.

Treatments	ALT (IU/L)	AST (IU/L)	ALP(IU/L)
Control (−)	48.16 ± 3.68a	37.46 ± 1.52a	89.34 ± 2.66a
Control (+)	66.74 ± 3.51c	68.52 ± 3.63c	342 ± 3.04c
100 mg/kg^bw^	59.62 ± 2.66bc	61.74 ± 3.42bc	247 ± 2.54bc
200 mg/kg^bw^	56.83 ± 3.11bc	36.65 ± 1.60a	213 ± 2.61b
300 mg/kg^bw^	53.75 ± 2.67 ab	34.73 ± 1.46a	175 ± 2.48b
400 mg/kg^bw^	48.49 ± 3.65a	30.85 ± 1.55a	140 ± 2.74b
PCM + 100 mg/kg^bw^	60.55 ± 3.42bc	48.22 ± 2.34b	276 ± 2.10bc
PCM + 200 mg/kg^bw^	57.46 ± 2.64bc	46.37 ± 2.21b	242 ± 2.45b
PCM + 300 mg/kg^bw^	55.34 ± 2.46bc	46.18 ± 2.54b	236 ± 2.62b
PCM + 400 mg/kg^bw^	50.61 ± 2.73b	43.25 ± 1.86 ab	205 ± 2.18b

Values are mean ± standard deviations of triplicate determination.

Values with different superscript (a–c) per column are significantly different.

PCM = Paracetamol 1 g/kg^bw^ thrice daily at 8-h interval.

**Table 5:** In vivo Antioxidant potentials of H. opposita extracts on treated and toxicant induced mice.

Treatments	Lipid Peroxidation (μM/g)	Catalase (μM/g)	Glutathione (μM/g)
Control (−)	96.85 ± 5.34^a^	66.43 ± 5.30^a^	31.66 ± 2.16^a^
Control (+)	139.38 ± 1.24^c^	44.31 ± 2.56^c^	17.40 ± 2.06^c^
100 mg/kg^bw^	130.65 ± 1.06^c^	42.36 ± 2.65^c^	20.15 ± 1.53^b^
200 mg/kg^bw^	124.42 ± 1.12^b^	49.23 ± 2.17^c^	22.36 ± 1.11^b^
300 mg/kg^bw^	121.36 ± 1.26^b^	52.46 ± 1.28^b^	23.32 ± 2.31^b^
400 mg/kg^bw^	117.35 ± 2.24^bc^	54.37 ± 1.16^b^	24.30 ± 1.62^b^
PCM + 100 mg/kg^bw^	121.63 ± 1.17^b^	56.28 ± 2.34^b^	25.14 ± 1.24^b^
PCM + 200 mg/kg^bw^	115.11 ± 2.13^bc^	57.12 ± 2.13^b^	25.23 ± 2.11^b^
PCM + 300 mg/kg^bw^	103.32 ± 1.65^b^	59.33 ± 1.43^b^	26.19 ± 1.17^ab^
PCM + 400 mg/kg^bw^	100.18 ± 1.12^b^	60.30 ± 2.07^ab^	28.65 ± 1.21^ab^

Values are mean ± standard deviations of triplicate determination.

Values with different superscript (a–c) per column are significantly different.

PCM = (Paracetamol) 1 g/kg^bw^ thrice daily at 8-h interval.

## References

[1] Maheswari C Maryaammal R Venkatunarayanan R Hepatoprotective activity of *Orthosiphopn stamineus* on liver damage caused by paracetamol in rats Jordan J Biol Sci 2008 1 3 105 8

[2] Mohamed Saleem TS Madhusudhana Chetty S Ramkanth S Rajan VST Mahesh Kumar K Gauthaman K Hepatoprotective herbs – a review Int J Res Pharm Sci 2010 1 1 1 5

[3] Oyagbemi AA Odetola AA Hepatoprotective effects of ethanol of *Cnidoscolus aconitifolins* on paracetamol-Induced hepatic damage in rats Pakistan J Biol Sci 2010 13 4 164 9 10.3923/pjbs.2010.164.169 20437682

[4] Aschale Y Wubetu M Abebaw A Yirga T Minwuyelet A Toru M A systematic review on traditional medicinal plants used for the treatment of viral and fungal infections in Ethiopia J Experiment Pharmacol 2021 13 807 15 10.2147/JEP.S316007 PMC8378932 34429665

[5] Jacob RA The integrated antioxidant system Nutr Res 1995 15 5 755 66

[6] Dressler S Schmidt M Zizka G Hoslundia. African plants – a photo guide Frankfurt/Main Forschungsinstitut Senckenberg 2014

[7] Sadri AS Antimalarial effect and other properties of *Hoslundia opposita* – a Review Glob J Pharm Sci 2017 4 3 555636 10.19080/GJPPS.2017.04.555636

[8] World Health Organization (WHO) General guidelines for methodologies on research and evaluation of traditional medicine Switzerland 2000

[9] The Organization of Economic Cooperation and Development (OECD) The OECD guideline for testing of chemical 420 Acute Oral Toxicity France 2010

[10] Lorke DA New approach to practical acute toxicity testing Arch Toxicol 1983 54 275 87 6667118 10.1007/BF01234480

[11] Doumas BT Watson WA Biggs HG Albumin standards and the measurement of serum albumin with bromocresol green Clin Chim Acta 1971 31 1 87 10.1016/0009-8981(71)90365-2 5544065

[12] Carroll JJ Roberta DHC Babson AL A simplified alkaline phosphotungstate assay for uric acid in serum Clin Chem 1971 7 158 60 10.1093/clinchem/17.3.158 5543187

[13] Hutson DH Pickering BA Donninger C Phosphoric acid triester–glutathione alkyltransferase. A mechanism for the detoxification of dimethyl phosphate triesters Biochem J 1972 127 1 285 93 10.1042/bj1270285 5073748 PMC1178583

[14] Fenech G Tommasini A Method of colorimetric determination of urea Boll Chim Farm 1952 91 10 391 5 13032247

[15] Watson D Rogers JA A study of six representative methods of plasma bilirubin analysis J Clin Pathol 1961 14 271 8 13783422 10.1136/jcp.14.3.271 PMC480210

[16] Abel LL Levy BB Brodie BB Kendall FE A simplified method for the estimation of total cholesterol in serum and demonstration of its specificity J Biol Chem 1952 195 1 357 66 14938387

[17] Lustgarten JA Wenk RE Simple, rapid, kinetic method for serum creatinine measurement Clin Chem 1972 18 11 1419 22 4652842

[18] Van Der Sluis AA Dekker M Verkerk R Jongen WMF An improved, rapid *in-vitro* method to measure antioxidant activity. Application on selected flavonoids and apple juice J Agric Food Chem 2000 48 4116 22 10995324 10.1021/jf000156i

[19] Ellman GL Tissue sulphydryl groups Arch Biochem Biophy 1959 82 70 2 10.1016/0003-9861(59)90090-6 13650640

[20] Cohen G Dembiec D Marcus J Measurement of catalase activity in tissue extracts Ann Biochem 1970 34 30 8 10.1016/0003-2697(70)90083-7 5440916

[21] Köhler I Jenett-Siems K Kraft C Siems K Abbiw D Bienzle U Herbal remedies traditionally used against malaria in Ghana: bioassay-guided fractionation of micro-glossa pyrifolia (asteraceae) *Zeitschrift für naturforschung C* J Biosci 2002 57 11–12 1022 7 10.1515/znc-2002-11-1212 12562088

[22] Muhammad NO Akolade JO Usman LA Oloyede OB Haematological parameters of alloxan-induced diabetic rats treated with leaf essential oil of Hoslundia opposita (Vahl.) EXCLI J 2012 11 1 671 PMC4874321 27231470

[23] Adjei S Entsua-Mensah P Amponsah IK Baah MK Kwakye NAA Addae-Kyereme NYK Pharmacognostic and physicochemical studies of the leaves of *Hoslundia opposita* Vahl (Lamiaceae) J Pharmacogn Phytochem 2020 9 5 2996 3001 10.22271/phyto.2020.v9.i5ap.12795

[24] Akah PA Odo CI Hepatoprotective effect of the solvent fractions of the stem of *Hoslundia opposita* Vahl (Lamiaceae) against carbon tetrachloride- and paracetamol-induced liver damage in rats Int J Green Pharm 2010 4 1 54 8 10.4103/0973-8258.62159

[25] Shukla P Kumar R Raib AK Detection of minerals in green leafy vegetables using laser induced breakdown spectroscopy J Appl Spectrosc 2016 83 5 872 7

[26] Ülger TG Songur AN Çõrak O Çakõroğlu FP Role of vegetables in human nutrition and disease prevention Intech 2018 Chapter 2 7 32 10.5772/intechopen.77038

[27] João C João SD Imai S Vegetables consumption and its benefits on diabetes J Nutr Therapeut 2017 6 1 1 10

[28] Hemmige NN Abbey L Asiedu SK An overview of nutritional and anti-nutritional factors in green leafy vegetables Horticul Int J 2017 1 2 58 65

[29] Emeka EJI Osundiya AO Biochemical, haematotological and histological effects of dietary supplementation with leaves of *Gnetum africanus* Weiw. In paracetamol-induced hepatotoxicity in rats Int J Pharmacol 2010 6 6 872 9

[30] Hinson JA Roberts DW James LP Mechanisms of acetaminophen-induced liver necrosis Handb Exp Pharmacol 2010 196 369 405 10.1007/978-3-642-00663-0_12 PMC2836803 20020268

[31] Oyedeji KO Bolarinwa AF Ojeniran SS Effect of paracetamol (acetaminophen) on haematological and reproductive parameters in male albino rats Res J Pharmacol 2013 7 21 5 10.36478/rjpharm.2013.21.25

[32] Miloš MM Marija DM Milica GP Branka IO Andraš ŠŠ Zorica SS Paracetamol-induced changes of haematobiochemical and oxidative stress parameters in rat blood: protective role of vitamin c and β-glucan Kragujevac J Sci 2016 38 135 46

[33] Tafere GG Tuem AK Gebre AK Balasubramaniam R *In vitro* antioxidant and *in vivo* hepatoprotective activities of root bark extract and solvent fractions of *Croton macrostachyus* Hochst. Ex Del. (Euphorbiaceae) on Paracetamol-Induced liver damage in mice J Exp Pharmacol 2020 12 301 11 32982486 10.2147/JEP.S259081 PMC7493212

[34] Jen JJ Hanne HA Review on liver function test The Danish Hepatitis C 2002 http://home3.inet.tele.dk/omni/hemochromatosis_iron.htm

[35] Yahya F Mamat SS Kamarolzaman MFF Seyedan AA Jakius KF Mahmood ND Hepatoprotective activity of methanolic extract of *Bauhinia purpurea* leaves against Paracetamol-Induced hepatic damage in rats Evidence-Based Compl Alt Med 2013 636580.10 10.1155/2013/636580 PMC3703353 23853662

[36] Yousef MI Omar SAM El-guendi MI Abdelmegid LA Potential protective effects of quercetin and curcumin on paracetamol-induced histological changes, oxidative stress, impaired liver and kidney functions and haematotoxicity in rat Food Chem Toxicol 2010 48 3246 61 10.1016/j.fct.2010.08.034 20804811

